# Low-cost fine-tuning of SmolVLA with smoothness regularization and chunk-aware auxiliary supervision for language-conditioned manipulation

**DOI:** 10.3389/fnbot.2026.1890245

**Published:** 2026-10-01

**Authors:** Chenkai He

**Affiliations:** Tianjin University of Science and Technology, Tianjin, China

**Keywords:** action regularization, action-chunk supervision, embodied intelligence, LIBERO, parameter-efficient fine-tuning, robot learning, SmolVLA, vision-language-action model

## Abstract

Vision-Language-Action (VLA) models offer a unified interface for language-conditioned manipulation. This paper reports a negative result: a low-cost post-training recipe for SmolVLA with LoRA, a temporal smoothness regularizer, and chunk-aware auxiliary supervision from action-variation pseudo labels does not improve manipulation success under a 30,000-step budget (approximately 1/67 of the original 2,000,000-step SmolVLA recipe). Because the auxiliary classifier is used only during training and the runtime chunk length remains fixed at *k* = 8, we denote the configuration SmolVLA+LoRA+S&C (smoothness and chunk-aware auxiliary losses) rather than “adaptive.” We evaluate four LIBERO suites with a unified protocol of 500 episodes per cell (50 episodes × 10 task variants). Over three training seeds, the matched fixed-chunk *k* = 8 baseline reaches a four-suite macro success rate of 10.9%±1.1, whereas SmolVLA+LoRA+S&C reaches 3.5%±1.4; the pre-specified primary comparison shows significant deficits on LIBERO-Spatial (−16.4 percentage points, exact sign-flip permutation *p* = 0.004) and LIBERO-Object (−17.5 pp, *p* = 0.002), with matched-pairs rank-biserial effect sizes of −0.96 and −1.0. Matched component ablations isolate the smoothness loss as the harmful term (smoothness-only collapses performance; chunk-classifier-only approximately matches the baseline), and executed-action logs show the regularizer *triples* trajectory jerk (1.69 vs. 0.59 RMS)—the opposite of its intent. A training-free inference-time temporal-ensembling baseline, by contrast, more than doubles success on the two suites where the baseline is competent (Spatial 36.2 vs. 19.0%; Object 56.2 vs. 24.0%) at a 2.3 × step-rate cost, and a 10–100K budget sweep shows the S&C deficit never closes. We conclude that, for the tested configuration, budgets, and benchmark, the added action-side losses are not competitive with a well-tuned fixed chunk, and inference-time ensembling is the stronger use of the same compute.

## 1 Introduction

Recent progress in embodied artificial intelligence has been driven by the convergence of vision, language, and control. Earlier language-conditioned robot learning established that natural-language instructions can supervise manipulation policies beyond narrowly scripted tasks (Lynch and Sermanet, 2021; Jang et al., 2021). Subsequent work expanded this line through spatial transport models, multimodal prompting, and large-sequence decision models, including Transporter Networks, CLIPort, VIMA, Gato, and RoboCat (Zeng et al., 2020; Shridhar et al., 2021; Jiang et al., 2023; Reed et al., 2022; Bousmalis et al., 2024). Vision-Language-Action (VLA) systems such as RT-1, RT-2, PaLM-E, Octo, OpenVLA, and SmolVLA have pushed toward unified policies that map multimodal observations and language instructions directly to robot actions (Brohan et al., 2023a,b; Driess et al., 2023; Octo Model Team et al., 2024; Kim et al., 2024; Shukor et al., 2025).

### 1.1 Scope: action-side regularization on a DL VLA backbone

A Vision-Language-Action policy is a deep neural network that maps visual observations and language instructions to robot actions; the present work adopts the compact SmolVLA backbone (Shukor et al., 2025) and asks whether two auxiliary supervision signals—a temporal smoothness regularizer and a chunk-aware auxiliary classifier—added to the imitation-learning objective can improve manipulation performance at a constrained compute budget.

### 1.2 VLA usefulness in neurorobotics

VLA models are of interest for neurorobotic manipulation (NRM) because they enable language-mediated control of manipulators. Applications discussed in the NRM literature include tabletop object relocation, sorting, and tool-oriented manipulation, as well as potential future assistive uses such as task-oriented arm training or voice-guided activities of daily living (Raspopovic, 2021). These applications favor policies that execute temporally coherent action sequences. The present study is fully simulation-based and does not evaluate any neural interface, rehabilitation system, assistive user population, physical robot, or safety mechanism; NRM deployments of the studied technique remain a potential future application rather than an implication established here. Within this scope, the study asks whether training-time action-side supervision improves task success and temporal action quality at a small-lab compute budget.

### 1.3 Research gap and motivation

This progress has gone hand in hand with the emergence of large open datasets and standardized evaluation environments such as RoboTurk, BridgeData V2, DROID, Open X-Embodiment, RLBench, robomimic, and LIBERO (Mandlekar et al., 2018; Walke et al., 2023; Khazatsky et al., 2024; Padalkar et al., 2024; James et al., 2020; Mandlekar et al., 2022; Liu et al., 2023). LIBERO is selected for this study among the available benchmarks because it provides language-conditioned tasks, standardized demonstrations with consistent control frequency, and a suite-level transfer structure that matches the post-training question studied here. While RLBench (James et al., 2020) and robomimic (Mandlekar et al., 2022) also offer manipulation benchmarks, they do not provide the same combination of language conditioning and suite-level diversity. The goal is to isolate the effect of low-cost SmolVLA post-training rather than to claim cross-benchmark generalization. Even so, a gap remains between the ambitions of foundation-scale VLA research and the constraints faced by ordinary research labs. Temporal action modeling is still an open design problem. Action chunking improves efficiency and stability, but a fixed chunk size can be mismatched to phase-varying manipulation dynamics; related issues have been studied in ACT, Diffusion Policy, Behavior Transformer, and Decision Transformer (Zhao et al., 2023; Chi et al., 2023; Shafiullah et al., 2022; Chen et al., 2021).

This paper studies a practical question for low-cost VLA adaptation: *Does adding a smoothness regularizer and a chunk-aware auxiliary classifier to the training objective improve a SmolVLA+LoRA policy at a compute budget that is realistic for a small lab?* The author evaluates this question with a controlled 30,000-step study on LIBERO.

The contribution is four-fold. First, we formulate the smoothness penalty in a component-weighted form that respects the heterogeneous physical dimensions of end-effector actions, and we quantify the per-dimension contributions of the resulting penalty. Second, we specify the chunk-aware pseudo-label construction in full (frozen quantile thresholds, quantile-to-class mapping, tie handling, and sliding-window aggregation) and audit its statistical properties on the training data. Third, we report a controlled negative-result evaluation under a unified 500-episode-per-cell protocol: with the two pivotal configurations trained over three seeds, neither the full method nor its matched component ablations improve over the *k* = 8 fixed-chunk SmolVLA+LoRA baseline, and exact sign-flip permutation tests localize the deficit to the suites where the baseline is competent. Fourth, we characterize the failure mode with component-level loss decompositions, matched smoothness-loss ablations including a gripper-excluded variant, trajectory-smoothness measurements, compute-cost accounting, an inference-time temporal-ensembling comparator, and a 10–100K budget sweep. The results clarify the boundary between training-time chunk-aware representation learning and inference-time adaptive chunk scheduling, and they identify the compute regime in which this lightweight action-side supervision is not effective.

The rest of the paper is organized as follows. Section 2 summarizes related work in foundation policies, VLA models, temporal action modeling, and efficient adaptation. Section 3 provides a structured state-of-the-art summary. Section 4 defines the action-output path, smoothness loss, pseudo-label construction, and training objective. Section 5 describes the compute budget, datasets, and evaluation protocol. Sections 6 and 7 present the empirical results and limitations.

## 2 Related work

### 2.1 Foundation policies and vision-language-action models

Generalist policy learning has evolved from early language-conditioned imitation (Lynch and Sermanet, 2021; Jang et al., 2021) toward foundation-style multimodal control (Reed et al., 2022; Bousmalis et al., 2024; Brohan et al., 2023a,b; Driess et al., 2023). Open-source efforts such as Octo, OpenVLA, and SmolVLA (Octo Model Team et al., 2024; Kim et al., 2024; Shukor et al., 2025) are especially relevant because they shift the conversation from proprietary capability to reproducible research infrastructure. The proposed method is positioned in this open-source VLA line.

### 2.2 Action chunking and temporal smoothing

ACT introduced chunked action prediction with a transformer policy to combat error compounding in imitation learning (Zhao et al., 2023). Diffusion Policy extended the idea to diffusion-based action generation (Chi et al., 2023); Behavior Transformer cast the same problem as sequence clustering (Shafiullah et al., 2022). The present paper is closest to this action-modeling line. Inference-time smoothing is a long-standing practical trick in this literature, including temporal ensembling of overlapping chunks (Zhao et al., 2023) and low-pass post-processing of action outputs. This study includes a training-side smoothness loss and identifies an inference-time ensemble baseline as a necessary comparator, so that the value of the training-time regularization can be judged against the simpler inference-time alternative once both are measured.

### 2.3 Efficient adaptation

LoRA (Hu et al., 2022) and representation learning work such as R3M (Nair et al., 2022) reduce downstream data and compute demands. LoRA is used throughout this study.

### 2.4 Tactile and haptic sensing as a future extension

Tactile and haptic sensing can enrich VLA policies for contact-rich NRM tasks—for example, grip-force adaptation from detected slip (Zhu et al., 2026). The present fully autonomous pipeline, however, uses only the vision and proprioception modalities exposed by LIBERO, so tactile sensing is treated as a natural future extension and haptic (human-in-the-loop) feedback is outside the scope of the current policy-learning framework.

## 3 State-of-the-art summary

Table 1 provides a compact positioning of VLA-related work for NRM-oriented manipulation, while Table 2 provides a structured summary of representative VLA-related work for language-conditioned manipulation. For each study, the table lists the author reference, year, methodology, materials (including sample size where reported), and methods.

**Table 1:** Compact positioning of VLA-related work for NRM-oriented manipulation.

Theme	Representative role in the literature	Relation to this paper
Language-conditioned imitation	Maps instructions and observations to manipulation actions	Provides the basic task interface studied here
Foundation VLA policies	Scale multimodal policy learning across tasks and datasets	SmolVLA is the open-source backbone adapted in this study
Temporal action modeling	Uses chunking, smoothing, or sequence prediction to reduce compounding errors	This paper tests training-side smoothness and chunk-aware supervision
NRM sensing extensions	Adds tactile or haptic feedback for contact-rich manipulation	Treated as future work because LIBERO provides visual/proprioceptive simulation observations only

**Table 2:** State-of-the-art summary of VLA-related work for language-conditioned manipulation.

Category	Author	Methodology	Materials	Methods
Foundation VLA policies				
Foundation VLA	Brohan et al. (2023b)	Large-scale transformer for real-world manipulation	130K real episodes, Everyday Robots	Transformer policy, FiLM conditioning
Foundation VLA	Brohan et al. (2023a)	Web-scale VLA: VLM co-fine-tuned with robot data	WebLI + robot demonstrations	PaLI-X/PaLM-E backbone, action tokenization
Foundation VLA	Octo Model Team et al. (2024)	Open-source generalist policy, multi-dataset pre-training	Open X-Embodiment (1M+ episodes)	Transformer, diffusion head, flexible observation
Foundation VLA	Kim et al. (2024)	Open-source VLA: Prismatic VLMs + discrete actions	Open X-Embodiment (970K episodes)	Prismatic-7B, discrete action bins, LoRA
Foundation VLA	Shukor et al. (2025)	Compact VLA for affordable robotics, 250M params	OXE + bridge, 2M training steps	SmolVLM backbone, discrete action tokenizer, LoRA
Temporal action modeling				
Action modeling	Zhao et al. (2023)	Chunked action prediction for fine-grained bimanual tasks	Custom bimanual teleoperation (6.5K episodes)	Transformer, CVAE-style, temporal ensembling
Action modeling	Chi et al. (2023)	Diffusion-based visuomotor policy, action-sequence generation	RoboTurk, Push-T, custom tasks (~200 demos)	CNN/Transformer diffusion, receding horizon
Benchmark, sensing, and calibration context				
Benchmark	Liu et al. (2023)	Lifelong learning benchmark for knowledge transfer	4 suites × 10 tasks × 50 demos	Behavior cloning, policy transfer, suite-level eval
VLA sensing survey	Ud Din et al. (2026)	Sensor modality and multi-modal fusion survey for VLA models	Literature survey covering VLA sensor fusion	Taxonomy of sensor modalities and fusion strategies
Calibration	Kim and Ha (2023)	Automatic extrinsic camera–LiDAR calibration via point-line correspondences	2D LiDAR, RGB camera, urban scenes	Targetless extrinsic parameter optimization
Calibration	Furgale et al. (2013)	Unified spatiotemporal calibration for multi-sensor robot systems	Hand-held sensor rig, diverse environments	B-spline continuous-time batch estimation
Action-side reg.	This study (2026)	Low-cost ( ≤ 30K steps) action-side supervision; negative result with component ablations	LIBERO 4 suites, SmolVLA+LoRA, 3 seeds	Component-weighted smoothness, sliding-window chunk-aware auxiliary classifier

Each related study is listed in author-reference format with methodology, materials (including sample size where reported), and methods. The present study is distinguished by its focus on low-cost ( ≤ 30K steps) action-side supervision with a compact VLA backbone and a multi-seed negative-result evaluation.

## 4 Materials and methods

### 4.1 Problem formulation and action-output space

Language-conditioned manipulation from offline demonstrations is considered. At time step *t*, the observation is *o*_*t*_ = {*I*_*t*_, *x*_*t*_, *l*} where *I*_*t*_ is the image stream, *x*_*t*_ the proprioceptive state, and *l* the task instruction. A VLA policy learns π_θ_:*o*_*t*_↦*a*_*t*_.

#### 4.1.1 Action-output space and gradient path

The action-output path used in this paper is clarified as follows. The SmolVLA backbone (Shukor et al., 2025) uses a discrete action tokenizer that bins each of the seven action dimensions (three translational, three rotational, one gripper) into 256 discrete bins. At training time, the model predicts a categorical distribution over bins per dimension and per step; the standard cross-entropy loss on these tokens constitutes $L_{\text{act}}$. The smoothness regularizer and chunk-aware auxiliary classifier, however, do *not* operate on the discrete token logits. They operate on the differentiable continuous action expectation â_*t*+*i*_, obtained by multiplying the bin centers by their softmax probabilities and summing, which yields a differentiable expectation of the bin centers with respect to the logits, as defined in Equation 1:


$$\begin{array}{l} {â}_{t+i}^{\left(d\right)}=\overset{256}{\underset{b=1}{\sum }}p_{b}^{\left(d\right)}\left(z_{t+i}\right)\cdot c_{b}^{\left(d\right)}, \end{array}$$ (1)

where $p_{b}^{\left(d\right)}$ is the softmax probability of bin *b* for dimension *d* and $c_{b}^{\left(d\right)}$ is the corresponding bin center. The expected-bin-center ${â}_{t+i}^{\left(d\right)}$ is differentiable in *z*_*t*+*i*_ through the softmax, so the smoothness penalty defined below propagates gradients back to the LoRA-adapted parameters of the action head.

Two decoding paths therefore coexist and are deliberately kept distinct. *Training-time regularization* operates on the differentiable expected bin center â, whereas *runtime execution* takes the discrete argmax bin per dimension, preserving the baseline decoding interface. These two paths can disagree: when the categorical distribution over bins is multi-modal or diffuse, the expectation can lie far from the argmax bin, so the smoothness penalty regularizes actions that differ from those actually executed at inference. We did not quantify this expectation–argmax mismatch in the present experiments, and it is an uncertainty of the study: part of the gap between regularized and executed behavior may originate here rather than from the smoothness objective itself.

### 4.2 Component-weighted smoothness regularization

#### 4.2.1 Heterogeneous physical dimensions

Let the action vector at step *t*. The action vector is defined in Equation 2, and the component-weighted smoothness regularizer and weighting matrix are given in Equations 3, 4.


$$\begin{array}{l} a_{t}={\left[\Delta x,\Delta y,\Delta z,\Delta r_{x},\Delta r_{y},\Delta r_{z},g\right]}^{⊤}\in {\mathbb{R}}^{7}, \end{array}$$ (2)

concatenating translational displacements, rotational displacements, and a gripper command. The unweighted Euclidean penalty used in the original formulation is replaced by a component-weighted penalty


$$\begin{array}{l} {ℒ}_{smooth}=\overset{k-2}{\underset{i=0}{\sum }}\|D({â}_{t+i+1}-{â}_{t+i}){\|}^{2}, \end{array}$$ (3)

with a diagonal weighting matrix


$$\begin{array}{l} D=\text{diag}\left(\frac{1}{\sigma_{\Delta x}},\ldots ,\frac{1}{\sigma_{g}}\right), \end{array}$$ (4)

where σ_*d*_ is the per-dimension standard deviation of step-to-step action changes in the training split of each LIBERO suite, computed once and frozen. This normalization makes the penalty invariant to the raw physical units of translations (in meters), rotations (in radians), and the gripper command. The direction of the correction deserves care: an *unweighted* ℓ_2_ assigns *smaller* contributions to lower-amplitude dimensions, not larger ones; dividing by a small σ_*d*_ *increases* the contribution of low-variance dimensions and thereby amplifies them in the total penalty. In our training data the amplification is substantial: after normalization, the binary gripper command alone accounts for approximately 28% of the unweighted-average smoothness penalty, despite being a discrete open/close signal rather than a smooth motion dimension. This is a specific hazard, because penalizing gripper transitions as if they were continuous motion discourages the discrete close-and-lift sequence that grasping requires. Section 6.4 therefore reports a matched ablation that excludes the gripper dimension from the smoothness loss, together with the per-dimension penalty contributions. Even with this weighting, the penalty still degrades performance at the studied compute budget, as the ablations show.

### 4.3 Per-suite normalized, sliding-window aggregated chunk pseudo labels

#### 4.3.1 Pseudo-label specification

The action-change statistic, sliding-window aggregation, and chunk-classification loss are defined in Equations 5–7, respectively.

The chunk-aware auxiliary classifier consumes pseudo labels constructed from the demonstrations alone; the complete construction is as follows, and the statistics quoted were measured on the training split (pseudo-label audit, Section 6.4).

1. *Action-change statistic*. At step *t* the statistic is the same component-weighted norm used in $L_{\text{smooth}}$:
$$\begin{array}{l} s_{t}=|D\left(a_{t+1}-a_{t}\right){|}_{2}, \end{array}$$ (5)
  which is dimensionless and invariant to per-suite action scales. At sequence boundaries the final frame of each demonstration is padded by repeating *s*_*t*_, i.e., no truncation of interior frames occurs.
2. *Thresholds*. Per suite, the 25th, 50th, and 75th percentiles of *s*_*t*_ are computed *globally over the entire training split*, pooled across episodes, and frozen. On LIBERO-Spatial the resulting thresholds are (0.138, 0.250, 0.416) (other suites differ; the medians of the four suites span 0.24–0.26).
3. *Quantile-to-class mapping*. The frame-level label is the number of thresholds strictly below *s*_*t*_, implemented as a half-open bucketization (bucketize with right=False); values exactly equal to a threshold fall into the upper bucket. On the audited split the number of frames exactly equal to a threshold was zero, so tie handling does not affect the reported labels.
4. *Candidate set*. The four buckets map to the candidate chunk lengths K={1,2,4,8} by rank order (bucket *j* maps to the *j*-th smallest candidate).
5. *Sliding-window aggregation*. The frame-level labels are aggregated by a length-9 centered plurality,
$$\begin{array}{l} {\bar{c}}_{t}^{*}=\text{Plurality}\left({\left\{c_{j}^{*}\right\}}_{j=t-4}^{t+4}\right), \end{array}$$ (6)
  with ties at the window boundary resolved toward the smaller window prefix, i.e., toward earlier frames. The classifier target is the aggregated label ${\bar{c}}_{t}^{*}$ aligned one-to-one with the pooled hidden state *h*_*t*_ and with the first step of the predicted chunk.

#### 4.3.2 Audited label statistics

The resulting class distribution is approximately balanced (28.9%, 24.0%, 22.1%, 25.0% for *k* = 1, 2, 4, 8 on LIBERO-Spatial). Two audited properties qualify the intended role of the aggregation. First, the plurality filter does *not* preserve short transient phases: before aggregation, 79% of frame-level label runs are at most two frames long (median run length 1, 90th percentile 4), and the length-9 plurality shortens or removes many of these runs, in line with the standard behavior of majority-style filters. Any claim that the construction preserves contact transients is therefore incorrect, and we do not make it. Second, because thresholds are frozen from the training split but batches are sampled episodically, per-batch quantiles drift around the frozen values (batch-threshold standard deviation 0.023–0.046 across suites); recomputing thresholds per batch would flip 14.4% of frame labels. The frozen-threshold choice is thus a modeling commitment with measurable sensitivity.

The auxiliary classifier is a two-layer MLP over pooled hidden states with loss


$$\begin{array}{l} L_{\text{chunk}}=-\log p\left({\bar{c}}_{t}^{*}|h_{t}\right), \end{array}$$ (7)

trained jointly with the action head and not used at inference.

### 4.4 Overall objective and training-time scope

The final objective is given in Equation 8:


$$\begin{array}{l} L=L_{\text{act}}+\lambda_{\text{smooth}}L_{\text{smooth}}+\lambda_{\text{chunk}}L_{\text{chunk}}. \end{array}$$ (8)

#### 4.4.1 Training-time vs. runtime adaptivity, and naming

$L_{\text{chunk}}$ is a *training-time* chunk-aware representation signal. The auxiliary classifier is not used at inference time, and the runtime chunk length remains fixed at *k* = 8. The method is therefore *not* an adaptive-chunk policy, and throughout this paper we denote it **SmolVLA+LoRA+S&C** (fixed-(*k* = 8) SmolVLA+LoRA with smoothness and chunk-aware auxiliary losses); earlier drafts used the misleading name “adaptive_k8,” which is retired. Of the two inference-time comparisons flagged in prior discussion, temporal ensembling of overlapping fixed-chunk predictions in the spirit of ACT (Zhao et al., 2023) is *included* in the present evaluation as a matched baseline (Section 6.1), because it is the most direct and inexpensive comparator for a training-time smoothness regularizer. An oracle-chunk upper bound that selects k∈K at runtime from ground-truth phase labels remains future work.

### 4.5 Statistical testing procedures

The statistical unit is the *task-level success rate* within a suite (10 tasks), computed from 50 episodes per task. For the pivotal comparison the unit is the seed-averaged per-task rate over the three training seeds. All tests are two-sided.

#### 4.5.1 Primary (pre-specified) comparison

The comparison SmolVLA+LoRA+S&C vs. the matched fixed-chunk *k* = 8 baseline was pre-specified as the primary hypothesis. For each suite we report: (i) the *exact sign-flip permutation test*: with *n* = 10 paired differences *d*_*k*_, all 2^10^ = 1, 024 sign patterns are enumerated, and *p* is the probability that |mean(*s*⊙*d*)|≥|mean(*d*)| under uniform signs—a valid null-hypothesis test that requires no distributional assumption; (ii) a *recentered bootstrap* *p*-value: differences are centered at their observed mean before resampling (*B* = 10, 000), so the resampling distribution is anchored at the null value of zero; and (iii) a percentile bootstrap 95% confidence interval for the mean paired difference. The earlier submission's task-level bootstrap, which resampled the observed differences without centering, produced a distribution around the observed effect rather than around the null and was not a valid null-hypothesis test; it has been replaced throughout.

#### 4.5.2 Effect size

For paired task-level comparisons we report the *matched-pairs rank-biserial correlation*, $r_{\text{rb}}=\left(W^{+}-W^{-}\right)/\left(W^{+}+W^{-}\right)$ computed on signed ranks of the non-zero differences. This replaces Cliff's delta, which is an unpaired dominance measure. Zero differences are excluded from the ranks (Wilcoxon zero_method=~wilcox~) and the number of excluded zeros is reported with every test.

#### 4.5.3 Secondary (exploratory) comparisons

All other pairwise comparisons (vs. *k*∈{1, 2, 4} baselines, component ablations, gripper-excluded variant, and the temporal-ensembling baseline) are labeled exploratory. They use the Wilcoxon signed-rank test (Wilcoxon, 1945), computed exactly when no ties or zeros are present and *n* ≤ 25, and asymptotically otherwise (the mode used is reported per test), with *Holm correction* over the exploratory family (Demšar, 2006).

#### 4.5.4 Hierarchical uncertainty over seeds

Because task-level rates share a training seed, the pivotal comparison additionally reports a hierarchical bootstrap that resamples training seeds (with replacement) and, within each resample, tasks (with replacement), yielding a 95% CI for the mean paired difference that reflects both sources of variation.

#### 4.5.5 Friedman test

For multiple comparisons across the four chunk lengths (*k*∈{1, 2, 4, 8}), the non-parametric Friedman test (Friedman, 1937) treats chunk lengths as treatments and tasks as blocks per suite (Demšar, 2006); it is reported as descriptive, secondary evidence.

The mathematical definitions of all evaluation metrics and test statistics are consolidated in Appendix Equations a1–a7.

## 5 Experimental setup

### 5.1 Compute budget, hardware, and code

All 30,000-step training runs use batch size 32 and LoRA rank 64 (2.97M trainable parameters, 0.66% of the 450M-parameter backbone) on RTX-3090-class GPUs under a matched software stack [Python 3.12 (Python Software Foundation, Beaverton, OR, United States), PyTorch 2.6.0+cu124, LeRobot 0.5.1, Transformers 5.3.0, PEFT 0.19.1]. Per-update time is approximately 0.48 s for both the baseline and the full method (data loading dominates), giving roughly 4 GPU-hours per 30K-step configuration on the training hardware used for the seeded comparison; a 100K-step baseline run on the evaluation server took approximately 44 GPU-hours (0.59 steps/s) and serves as the upper end of the budget sweep. The 30,000-step budget is approximately 1/67 of the 2,000,000-step budget used by the original SmolVLA paper (Shukor et al., 2025); this constraint is a deliberate feature of the design, as the target use-case is a small-lab compute envelope. The backbone is the published lerobot/smolvla_base checkpoint as consumed by the archived training code; the code archive fixes the exact revision. An anonymized repository containing the training and evaluation code, the configuration for every table row, the checkpoint-selection rule (final checkpoint), data preprocessing, the σ_*d*_ computation, the pseudo-label implementation, simulator and evaluation seeds, raw per-task and per-seed outcomes, and the scripts producing every table and figure is provided during review.

### 5.2 Datasets and evaluation protocol

Training is conducted and evaluation is performed on four LIBERO suites (Liu et al., 2023): LIBERO-Spatial, LIBERO-Object, LIBERO-Goal, and LIBERO-10. LIBERO is selected because it provides language-conditioned manipulation tasks, standardized demonstrations, and suite-level transfer structure that match the post-training question studied here. RLBench and robomimic are important alternative benchmarks, but they are not used as the primary validation set because the goal is to isolate low-cost SmolVLA post-training under the LIBERO task family rather than to claim broad cross-benchmark generalization.

#### 5.2.1 Evaluation units (explicit)

Each suite contains 10 task variants, and every evaluated cell uses the same protocol: 500 episodes per suite-level cell = 50 episodes per task variant × 10 task variants. Per-task success rates are therefore computed from 50 rollouts each and change in increments of 2 percentage points. All rows of the main results table share this protocol. Episode totals per family: the pivotal pair (SmolVLA+LoRA+S&C and the *k* = 8 baseline) is evaluated over three training seeds, i.e., 3 × 4 × 500 = 6, 000 episodes per method (12, 000 total); the *k* = 1/2/4 baselines and the temporal-ensembling baseline contribute 6, 000 and 2, 000 episodes respectively; the component-ablation study (smoothness-only and chunk-only over two seeds, plus the gripper-excluded variant) contributes 9, 000 episodes; and the budget sweep contributes 6, 000 external checkpoint-evaluation episodes, for a total of 35, 000 evaluation episodes across the revision. Simulator initialization seeds are drawn from the task-specific initialization distribution with fixed seeds shared across methods, so paired comparisons see identical initial conditions.

#### 5.2.2 Train/test separation and seed usage

The LIBERO benchmark (Liu et al., 2023) provides a fixed set of 50 human-teleoperated demonstrations per task for training, with evaluation episodes drawn from the task-specific initialization distribution serving as a held-out test set, following the original LIBERO design. The pivotal configurations (SmolVLA+LoRA+S&C and its matched *k* = 8 baseline) are each trained over three independent seeds and reported as mean±sample standard deviation over seeds. A held-out validation split is not used for hyperparameter selection, which is acknowledged as a limitation in Section 8.

#### 5.2.3 Statistical reporting

For every cell we report the success rate $\hat{p}$, the Wilson 95% confidence interval given *n* = 500 episodes, and *n* itself. Inference follows Section 4.5: the pre-specified primary comparison uses the exact sign-flip permutation test with a recentered bootstrap CI and the matched-pairs rank-biserial effect size; all other comparisons are exploratory Wilcoxon tests with Holm correction.

### 5.3 Unified configuration and chunk-size convention

#### 5.3.1 Configuration consolidation

Table 3 consolidates the chunk-related quantities used in the experiments. There are two such quantities: (a) *k*_train_, the training- and inference-time action-prediction horizon (also called “chunk size” or *k*); and (b) |K|, the size of the candidate set used by the auxiliary classifier. The SmolVLA default *k* = 50 is not used in the LIBERO experiments; all evaluated fixed-chunk settings use *k*∈{1, 2, 4, 8}. Algorithm 1 summarizes the unified training and evaluation protocol.

**Table 3:** Unified training configuration.

Component	Setting
Backbone	SmolVLA base checkpoint (lerobot/smolvla_base)
Adaptation method	LoRA, rank 64
Optimizer learning rate	1 × 10^−4^
Warmup/decay/final LR	1,000/30,000/2.5 × 10^−6^
Batch size	32
Total optimizer steps	30,000 (≈1/67 of paper's 2 M)
Training/inference chunk size *k*	{1, 2, 4, 8}
Action-prediction horizon *n*_action_steps_	= *k* (matched)
Candidate set K	{1, 2, 4, 8} unless noted
Smoothness weight λ_smooth_	0.1 (default), ablated {0.2, 0.5, 1.0}
Chunk-supervision weight λ_chunk_	1.0 (default), ablated {0.5, 2.0}
Action tokenizer	256 bins per dimension
Action dimensions *d*	7 (Δ*x*, Δ*y*, Δ*z*, Δ*r*_*x*_, Δ*r*_*y*_, Δ*r*_*z*_, *g*)
Per-dim normalization σ_*d*_	per-suite std, frozen at preprocessing
Evaluation episodes per cell	500 (50 per task variant × 10 tasks), all cells
Total evaluation episodes	35,000 (this revision's rerun campaign)
Compute profile	RTX-3090-class 24 GB (training and evaluation)
Code and engineering files	Anonymized repository provided during review

The original submission reported inconsistent chunk-related quantities across tables and algorithm; the present table consolidates them.

Algorithm 1 Unified training and evaluation protocol. 
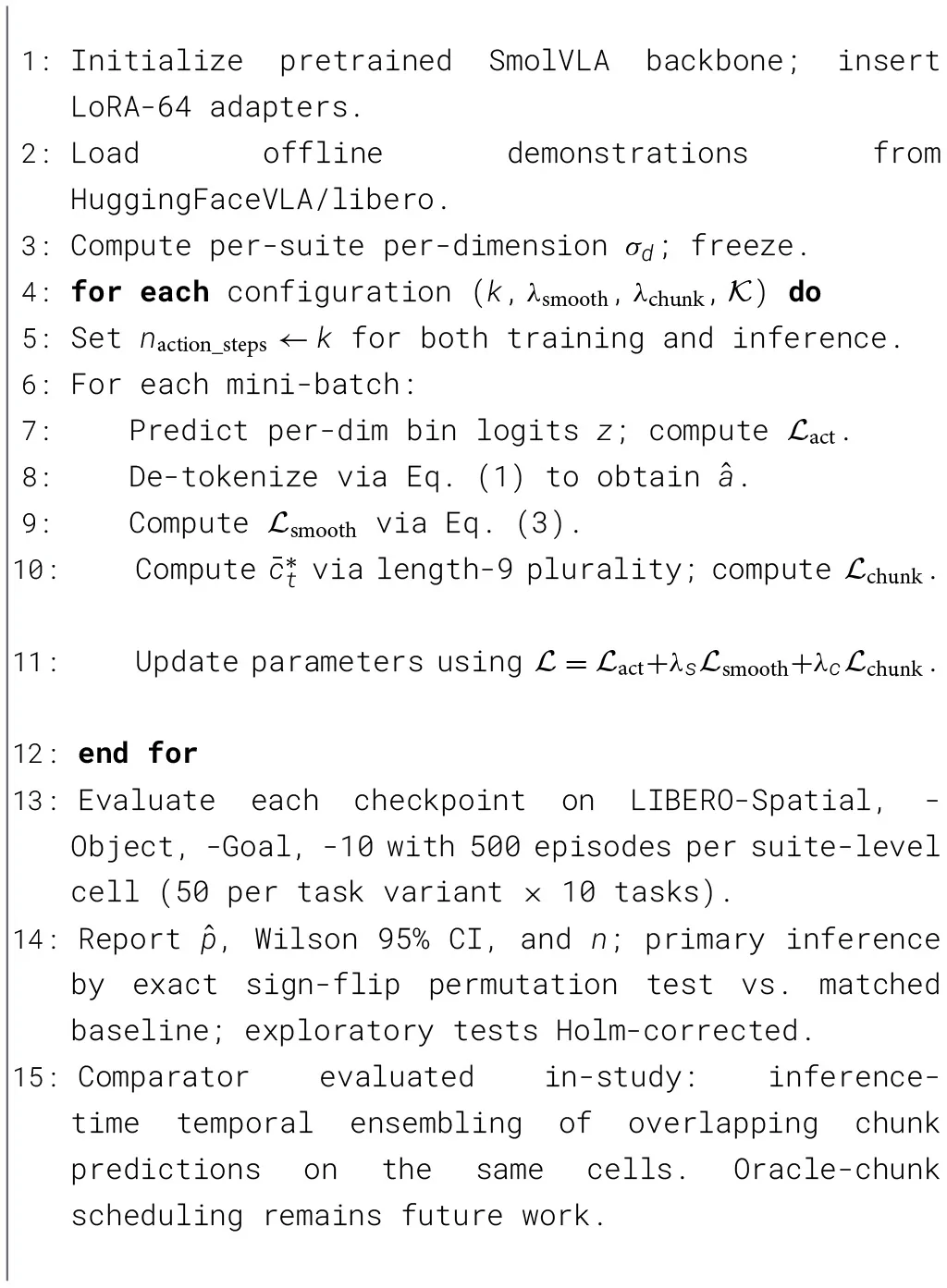

### 5.4 Research questions

The experiments ask four questions:

**RQ1 (primary)**. Does SmolVLA+LoRA+S&C improve over the matched fixed-chunk *k* = 8 SmolVLA+LoRA baseline at the studied 30,000-step budget?

**RQ2 (component attribution)**. Which of the two auxiliary losses is responsible for the outcome? Matched ablations train baseline + smoothness-only, baseline + chunk-classifier-only, and baseline + both.

**RQ3 (inference-time comparator)**. Does a training-free inference-time temporal ensemble of overlapping chunk predictions (Zhao et al., 2023) change baseline performance, and at what latency cost?

**RQ4 (budget dependence)**. Does the deficit narrow or widen across a 10–100K step budget sweep with checkpoint-level evaluation?

**Future comparison**. An oracle per-task chunk-selection upper bound over K={1,2,4,8} remains future work; it would quantify the headroom available to true runtime adaptive scheduling, which this training-time method does not attempt.

## 6 Results

### 6.1 Main results under the unified protocol

Table 4 reports the main comparison. Every cell is a suite-level success rate over 500 episodes (50 per task variant × 10 tasks). The pivotal rows (*k* = 8 baseline and SmolVLA+LoRA+S&C) are averaged over three training seeds and reported as mean ± sample standard deviation; remaining rows are single-seed (42).

**Table 4:** Main results on LIBERO under the unified protocol (500 episodes per suite-level cell = 50 per task × 10 tasks; success rate %).

Method	*k*	Spatial	Object	Goal	LIBERO-10	Macro-4
Panel A: fixed-chunk SmolVLA+LoRA baselines (no auxiliary losses), seed 42						
Fixed baseline	1	20.2	25.2	18.4	0.2	16.0
Fixed baseline	2	16.4	18.2	27.2	1.4	15.8
Fixed baseline	4	21.2	24.4	26.2	2.0	18.5
Panel B: pivotal comparison (mean ± SD over three training seeds)						
Fixed baseline	8	19.0 ± 3.0	24.0 ± 1.8	0.6 ± 0.2	0.0 ± 0.0	10.9 ± 1.1
SmolVLA+LoRA+S&C	8	2.6 ± 0.9	6.5 ± 4.7	4.3 ± 5.7	0.6 ± 0.7	3.5 ± 1.4
Panel C: inference-time comparator on the *k* = 8 baseline (training-free), seed 42						
Fixed baseline + TE	8	36.2	56.2	0.0	0.0	23.1

Pivotal rows: mean±sample SD over three training seeds (42, 123, 999); other rows: seed 42. Macro-4 is the unweighted mean over the four suites. TE= inference-time temporal ensembling applied to the k=8 baseline (training-free).

The four-suite macro success rate is 10.9%±1.1 for the matched *k* = 8 baseline vs. 3.5%±1.4 for SmolVLA+LoRA+S&C. The deficit is concentrated exactly where the baseline is competent: LIBERO-Spatial (19.0%±3.0 vs. 2.6%±0.9) and LIBERO-Object (24.0%±1.8 vs. 6.5%±4.7), whereas both configurations are near floor on LIBERO-Goal and LIBERO-10. The earlier submission's headline numbers (single-seed macros of 17.75 vs. 5.25%) came from a 100-episode-per-cell protocol with 10 episodes per task and are superseded by the present 500-episode matrix.

SmolVLA+LoRA+S&C is below the *k* = 8 baseline in all six suite–seed cells of the two suites where the baseline performs meaningfully (Spatial, Object). In the floor suites the ordering is mixed (e.g., Goal seed 999: 11.0 vs. 0.8%) and both methods are near zero, so no reliable ordering exists there; the auxiliary losses neither rescue the floor suites nor are they meaningfully worse in them.

### 6.2 Primary inference and exploratory comparisons

Table 5 reports inference under the procedures of Section 4.5. For the pre-specified primary comparison (SmolVLA+LoRA+S&C vs. the matched *k* = 8 baseline, seed-averaged per-task rates, 10 tasks per suite), the exact sign-flip permutation test rejects the null on LIBERO-Spatial [mean paired difference −16.4 percentage points, *p* = 0.0039, 95% bootstrap CI (−20.9, −11.3), matched-pairs rank-biserial *r*_rb_ = −0.96] and LIBERO-Object [−17.5 pp, *p* = 0.0020, CI (−21.7, −13.7), *r*_rb_ = −1.0]. On LIBERO-Goal (+3.7 pp, *p* = 0.19; two zero differences excluded) and LIBERO-10 (+0.6 pp, *p* = 0.50; eight zeros excluded) the differences are not significant and both methods are near floor. Recentered bootstrap *p*-values agree with the permutation tests. Hierarchical bootstrap over seeds and tasks yields CIs of (−21.9, −10.8) (Spatial) and (−23.2, −11.8) (Object), so the deficit is robust to training-seed variation.

**Table 5:** Primary (pre-specified) comparison: SmolVLA+LoRA+S&C vs. matched *k* = 8 baseline, seed-averaged per-task success rates (*n* = 10 tasks).

Suite	Mean diff. (pp)	Sign-flip *p*	95% CI (pp)	*r*_rb_	Zeros	Hier. CI (pp)
Spatial	−16.4	0.0039	−20.9, −11.3	−0.96	0	−21.9, −10.8
Object	−17.5	0.0020	−21.7, −13.7	−1.00	0	−23.2, −11.8
Goal	+3.7	0.19	−0.1, +10.1	+0.67	2	−0.4, +16.4
LIBERO-10	+0.6	0.50	0.0, +1.5	+1.00	8	0.0, +2.1

Sign-flip *p*, exact enumeration of 2^10^ patterns; CI, percentile bootstrap (10,000 resamples) for the mean paired difference; Hierarchical CI, bootstrap resampling seeds then tasks; Zeros, zero differences excluded from ranks/tests.

#### 6.2.1 Exploratory family

All other pairwise comparisons against the *k* = 8 baseline (seed 42 cells: *k*∈{1, 2, 4} baselines, smoothness-only and chunk-only ablations, the gripper-excluded variant, and the temporal-ensembling baseline; 24 tests in total) are Wilcoxon signed-rank tests with Holm correction over the family; the full table with per-test mode, zero counts, and adjusted *p*-values is emitted by the released statistics script (STATS_V6.json). Several contrasts reach raw *p*∈(0.004, 0.008) with large effect sizes (|*r*_rb_|≥0.93): smoothness-only is far below the baseline on Object (−25.0 pp) and Spatial (−18.8 pp); the temporal ensemble is far above it on Object (+30.8 pp) and Spatial (+17.2 pp); the gripper-excluded variant remains below on Spatial (−11.4 pp). After Holm correction over the 24-test family, however, no exploratory *p* survives at 0.05 (minimum adjusted *p* = 0.10), so we report these as strong, consistent directional evidence rather than confirmed differences. Chunk-only is statistically indistinguishable from the baseline everywhere except a mild Spatial deficit (−5.4 pp, raw *p* = 0.07). The Friedman test across chunk lengths is retained as descriptive evidence only.

### 6.3 Component ablations, gripper exclusion, and additional comparisons

#### 6.3.1 RQ2 (matched component ablation)

Table 6 isolates the two auxiliary losses under the unified protocol (seed 42, 500 episodes per cell): baseline, baseline+smoothness-only, baseline+chunk-classifier-only, and baseline+both, all at the same chunk size, budget, and evaluation protocol. The attribution is unambiguous: *the smoothness loss is the harmful term*. Smoothness-only collapses performance on the suites where the baseline is competent (Spatial 0.2%, Object 0.4%), chunk-only approximately matches the baseline (Object 25.2 vs. 25.4%; Spatial 13.6 vs. 19.0%), and the full combination sits close to the smoothness-dominated level. No single-loss variant improves over the matched baseline.

**Table 6:** Matched component ablation at 30K steps (seed 42, 500 episodes per cell, success rate %).

Configuration	Spatial	Object	Goal	LIBERO-10	Macro
Baseline (*k* = 8, no aux. losses)	19.0	25.4	0.6	0.0	11.3
+ smoothness loss only (λ_*s*_ = 0.1)	0.2	0.4	1.0	1.4	0.8
+ chunk-classifier loss only (λ_*c*_ = 1.0)	13.6	25.2	0.4	0.0	9.8
+ both (SmolVLA+LoRA+S&C)	3.6	12.0	1.0	1.4	4.5
S&C with gripper excluded from smoothness	7.6	19.8	—	—	—

“Both” is the full SmolVLA+LoRA+S&C configuration. The gripper-excluded row removes only the gripper dimension from the smoothness penalty.

#### 6.3.2 Gripper exclusion

Section 4.2 identifies the gripper command as an amplification hazard of the σ_*d*_ normalization (28% of the penalty from a binary dimension). Excluding the gripper dimension from the smoothness penalty alone recovers a substantial part of the deficit (Spatial 7.6 vs. 3.6%; Object 19.8 vs. 12.0%) while remaining below the baseline, consistent with over-regularization concentrated in, but not limited to, the gripper dimension.

#### 6.3.3 RQ3 (inference-time comparator)

The training-free temporal-ensembling baseline (exponentially weighted average of overlapping chunk predictions, recomputed every step) is reported in Table 4 Panel C. It *more than doubles* success on both suites where the baseline is competent—Spatial 36.2 vs. 19.0% (+17.2 pp, raw *p* = 0.006, *r*_rb_ = 0.93) and Object 56.2 vs. 24.0% (+30.8 pp, raw *p* = 0.004, *r*_rb_ = 0.96)—while leaving the floor suites unchanged (Goal 0.0%, LIBERO-10 0.0%), for a four-suite macro of 23.1% vs. the baseline's 10.9%. The comparison the reviewer requested is therefore decisive in this regime: a zero-training, inference-time smoother delivers the largest gains in the study at a 2.3 × step-rate cost (Table 7), whereas the training-time smoothness regularizer loses 16–17 pp *and* degrades measured trajectory smoothness (Section 6.6).

**Table 7:** Trajectory smoothness (executed-action logs, LIBERO-Spatial+Object, seed 42, 500 episodes per suite per configuration; mean ± SD over episodes) and compute cost.

Metric	Baseline *k* = 8	S&C	S&C (gripper excl.)
Normalized action variation	0.39	0.91	0.64
Jerk RMS (translation)	0.58	1.66	1.65
Jerk RMS (rotation)	0.11	0.28	0.28
Total jerk RMS	0.59 ± 0.07	1.69 ± 0.09	1.68 ± 0.10
Gripper variation per episode (Σ|Δ*g*|)	66.3 ± 30.7	182.8 ± 31.2	70.3 ± 25.5
Control reversals per episode	741	1054	1021
Rollout step rate, Spatial (steps/s)	4.39	—	—
Rollout step rate, Spatial + TE	1.91 (2.3 × slower)	—	—
Rollout step rate, Object (steps/s)	5.61	—	—
Rollout step rate, Object + TE	2.35 (2.4 × slower)	—	—
Peak GPU memory, training (GB)	7.3	≈7.3	—

S&C increases every smoothness-disorder metric relative to the baseline; excluding the gripper dimension restores gripper variation to the baseline level but not overall jerk. Step rates are medians over ~70,000 logged rollout steps per configuration on the same GPU.

#### 6.3.4 Coefficient sweeps (secondary)

Coefficient sweeps over λ_smooth_∈{0.2, 0.5, 1.0} and λ_chunk_∈{0.5, 2.0} and reduced candidate sets were run under the earlier 100-episode-per-cell protocol; no sweep direction improved over the matched baseline, and the results did not follow a monotone pattern in λ_smooth_. Because these sweeps predate the unified protocol, we do not tabulate them alongside the matched ablations; the raw outcomes are archived in the released evaluation records. We therefore do not claim a monotone degradation relationship, in contrast to the earlier submission.

### 6.4 Diagnostic analysis of the negative outcome

**(i) The primary objective is measurably impaired**. Because the S&C total loss contains extra terms, comparing totals across methods is uninformative; Figure 1 therefore decomposes the objective. In the matched 20K-step pair, the S&C action-token loss plateaus at ≈0.83 vs. ≈0.56 for the baseline, so the auxiliary terms do impair optimization of the primary head itself—not merely add constant offsets to a reported total. This is consistent with the ablation attribution: the smoothness gradient (loss ≈1.6 at plateau, weighted 0.16 into the total) competes with action prediction rather than acting as a benign regularizer at this budget.

**Figure 1:**
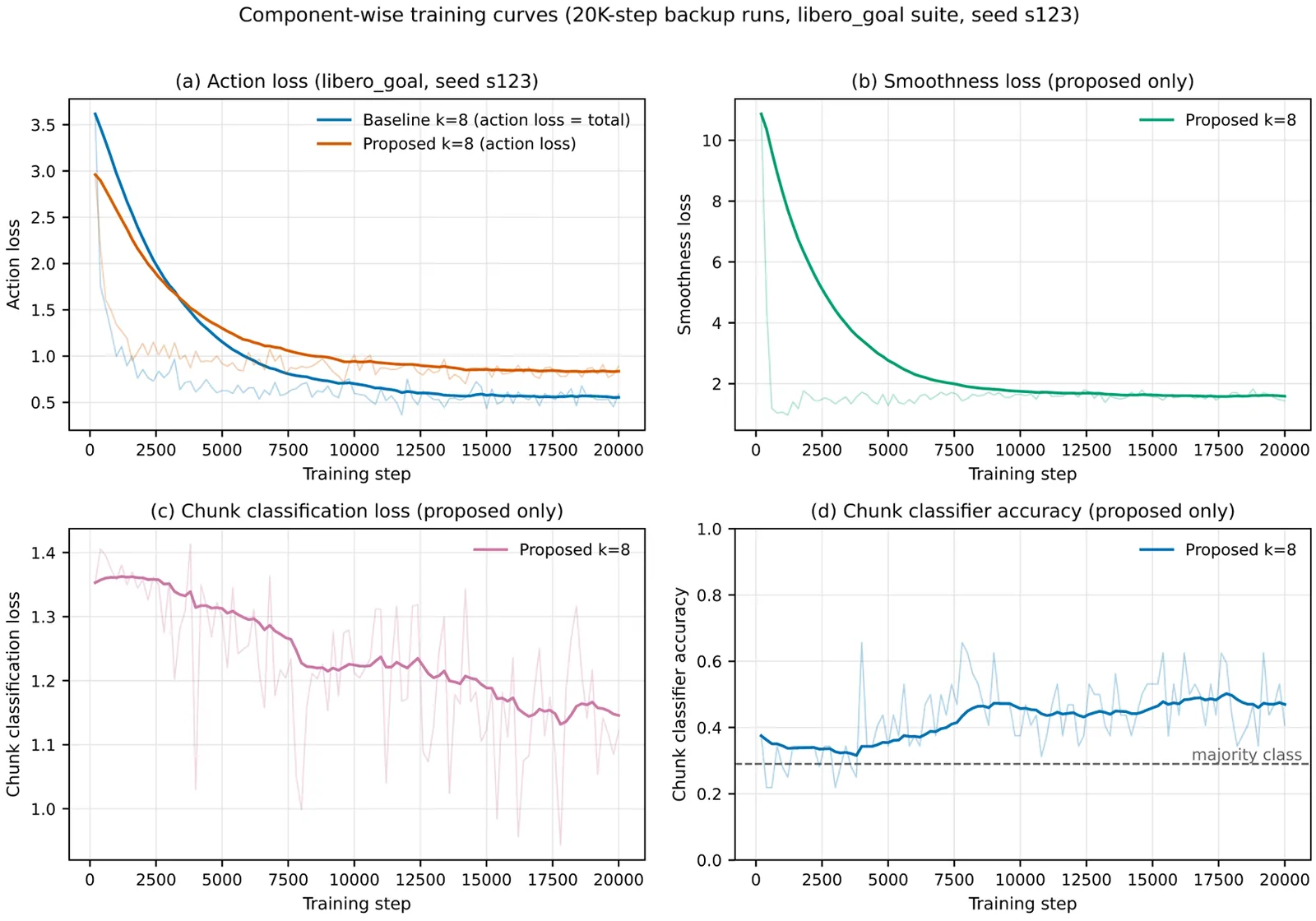
Training diagnostics from logged component losses (matched 20K-step pair, LIBERO-Goal seed 123; EMA-smoothed; other matched pairs in the released records). **(a)** Action-token loss $L_{\text{act}}$: the S&C configuration plateaus at ≈0.83 vs. ≈0.56 for the baseline, so the auxiliary terms impair the primary objective itself. **(b)** Smoothness loss $L_{\text{smooth}}$ (S&C only; plateau ≈1.6, i.e., weighted contribution ≈0.16 at λ_*s*_ = 0.1). **(c)** Chunk-classification loss $L_{\text{chunk}}$ (S&C only). **(d)** Chunk-classifier top-1 accuracy with two reference lines: the majority-class baseline of the pseudo-label distribution (0.29) and the uniform-random baseline (0.25).

**(ii) Classifier convergence, measured against the right baseline**. The chunk-classifier top-1 accuracy saturates between 0.41 and 0.47 across the matched runs by 20K steps. The relevant reference is the majority-class rate of the pseudo-label distribution itself (0.29; logged majority-following accuracy ≈0.30), not only the uniform rate (0.25); the classifier exceeds the majority baseline by roughly 12–18 percentage points, so it learns *some* signal but remains far from the oracle, consistent with pseudo labels whose short-run structure (median frame run length 1; 79% of runs ≤ 2 frames) is difficult to predict from pooled hidden states.

**(iii) Over-regularization, localized by ablation**. The attribution follows from Table 6 rather than from loss-level intuition: smoothness-only reproduces the collapse, chunk-only does not, and excluding the single gripper dimension from the smoothness penalty recovers several percentage points. Together with the per-dimension penalty accounting (gripper ≈28% of the penalty), the evidence localizes the failure to the smoothness term's interaction with discrete, low-variance action dimensions. These attributions remain ablation-based: we do not claim gradient-level mechanisms, and gradient-norm or gradient-conflict measurements are future work.

### 6.5 Rollout illustration

Figure 2 shows the pseudo-label construction regenerated from the implemented algorithm; Figure 3 is a schematic of the auxiliary branch; Figure 4 summarizes the data flow; Figure 1 shows the component-loss diagnostics.

**Figure 2:**
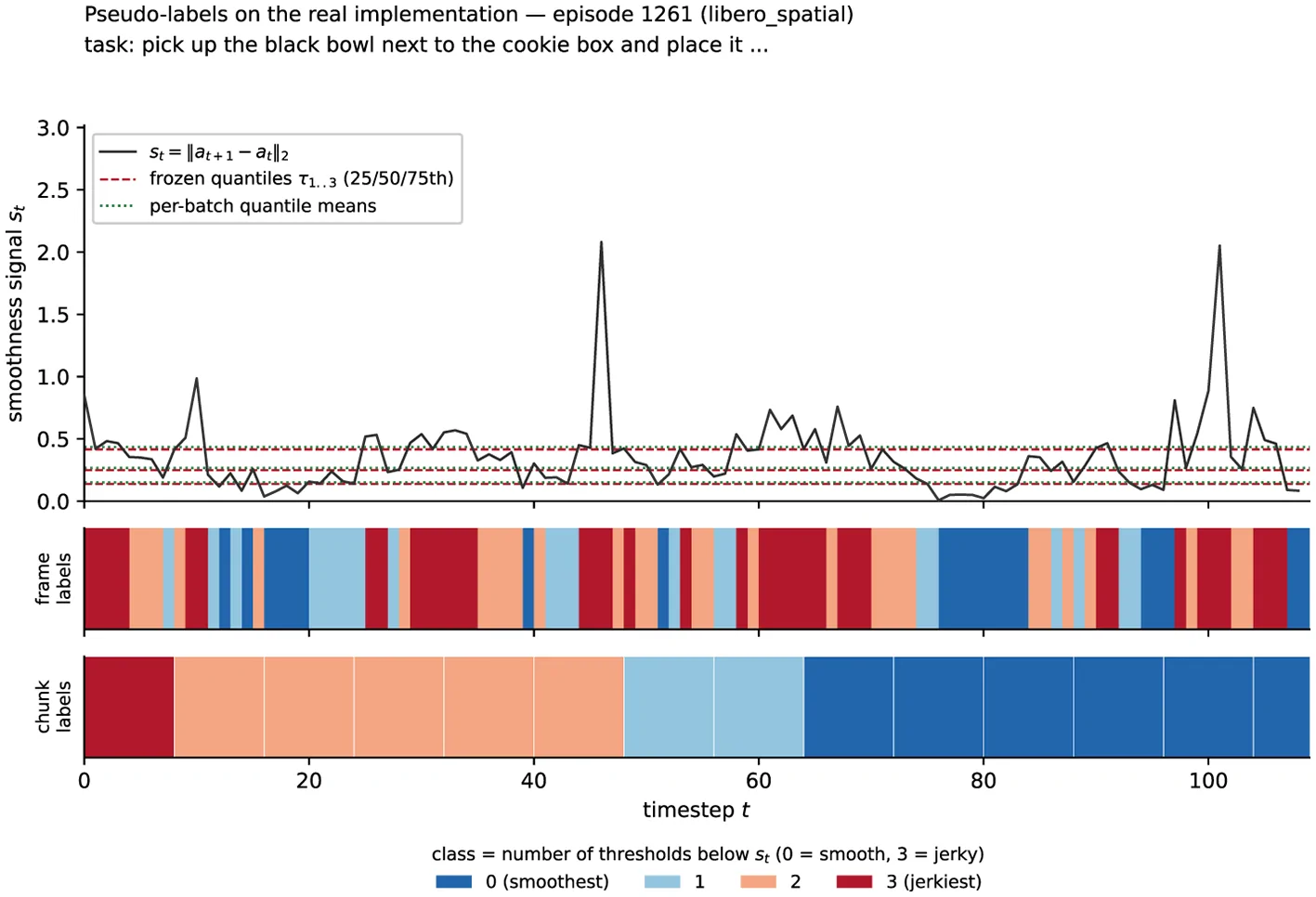
Pseudo-label construction, regenerated directly from the implemented algorithm on the LIBERO-Spatial training split: per-dimension normalized action-change statistics *s*_*t*_ are partitioned by frozen quantile thresholds (25/50/75th percentiles: 0.138/0.250/0.416) and aggregated by a length-9 sliding-window plurality into chunk pseudo labels ${\bar{c}}_{t}^{*}$. Every label shown is produced by the released code path; the resulting class distribution is 28.9%, 24.0%, 22.1%, 25.0% for *k* = 1, 2, 4, 8.

**Figure 3:**
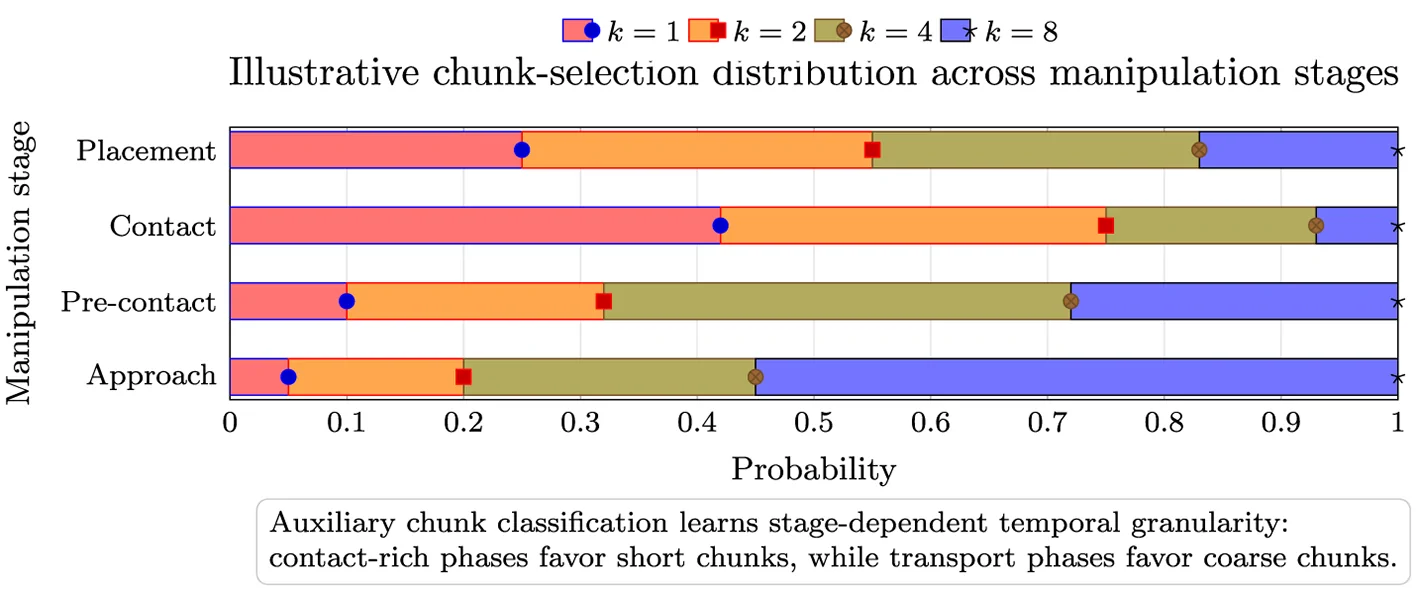
Schematic of the auxiliary chunk-classifier branch (conceptual illustration, not measured data): the classifier is trained on pooled hidden states and used only during training; the runtime decoding interface remains fixed-chunk.

**Figure 4:**
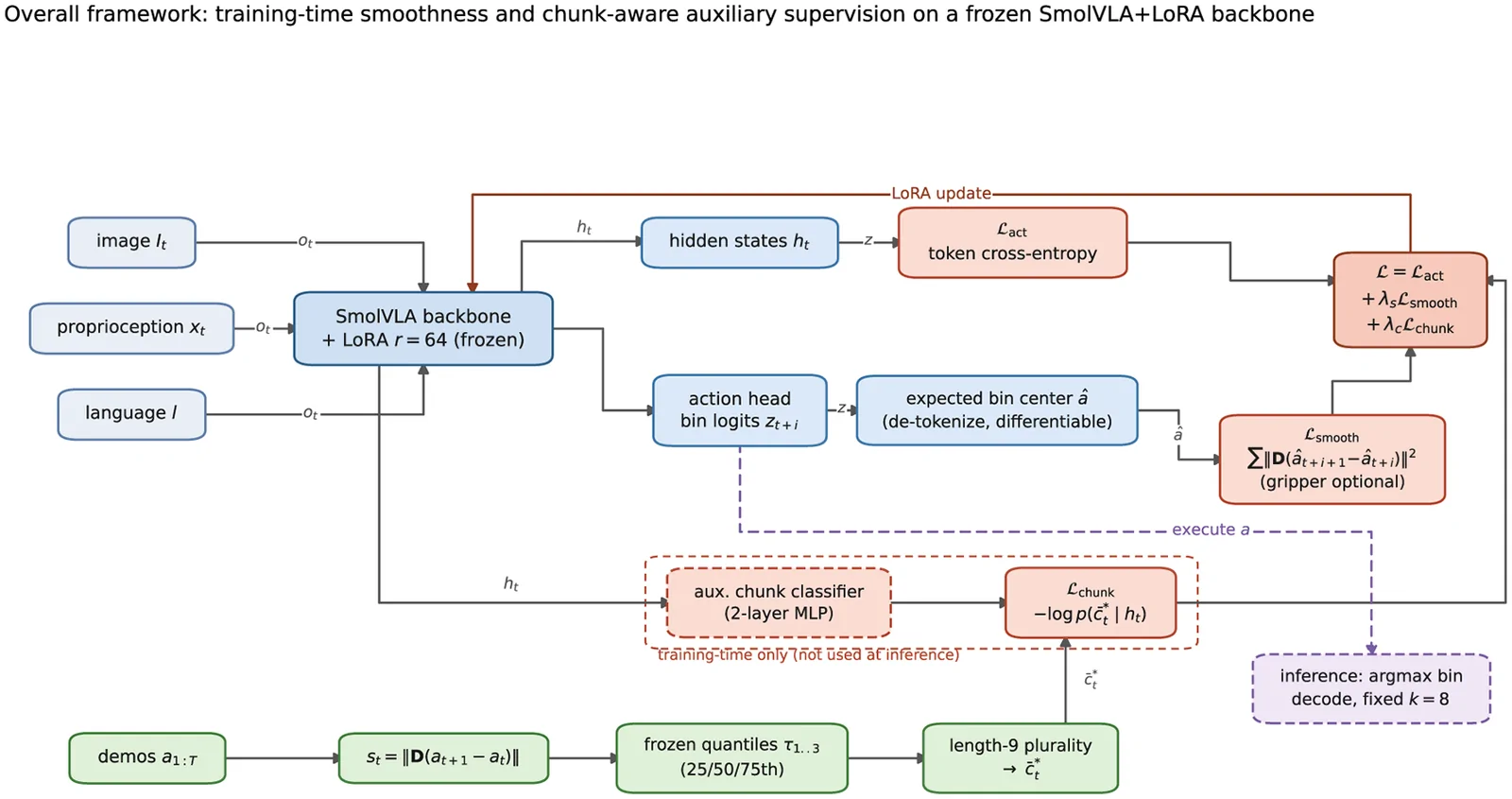
Overall framework (schematic; node sizes are auto-fitted to labels, arrows follow orthogonal routes). The SmolVLA backbone and LoRA adaptation path are unchanged. Smoothness regularization and chunk-aware auxiliary supervision operate on the de-tokenized continuous action expectations â and pooled hidden states *h*_*t*_ respectively; the dashed enclosure marks training-only components.

Figure 5 shows *one illustrative rollout pair* from the evaluation set. We do not use it to establish a failure-mode frequency: no failure taxonomy was annotated across the evaluation episodes, so quantitative failure-mode statistics are not claimed anywhere in this paper.

**Figure 5:**
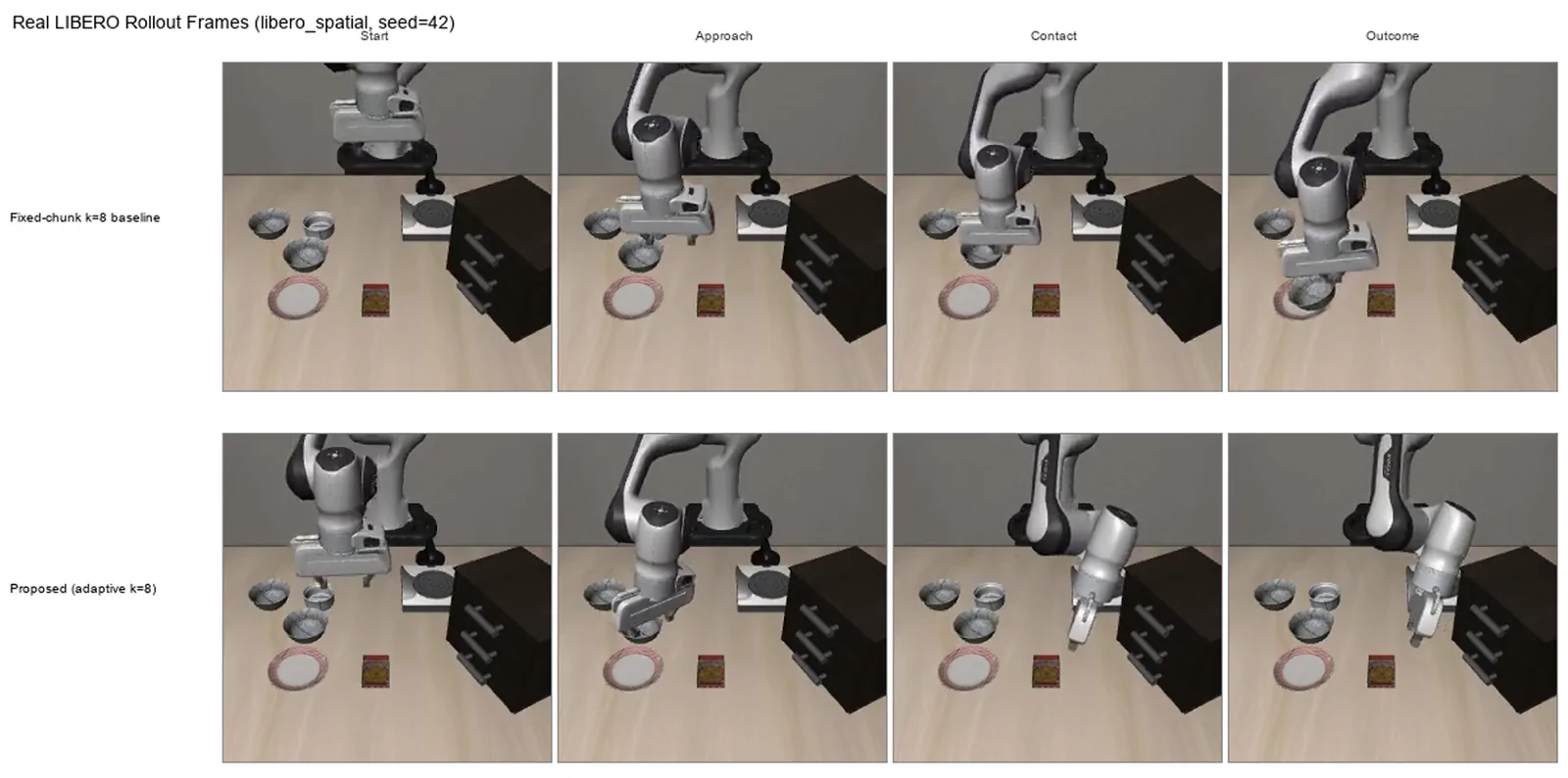
One illustrative rollout pair from LIBERO-Spatial (seed 42, task 0, episode 0). **Top:** the fixed-chunk *k* = 8 baseline completes the pick-and-place sequence. **Bottom:** the S&C configuration stalls during the approach phase and does not grasp. Raw simulator camera frames; shown as a qualitative example, not as evidence of a quantified failure mode.

### 6.6 Trajectory smoothness and compute cost

A paper that regularizes smoothness must report smoothness outcomes. From dedicated evaluation runs that log the full executed action sequences (500 episodes per suite per configuration on LIBERO-Spatial and LIBERO-Object, seed 42), Table 7 reports normalized action variation, translation- and rotation-specific jerk, gripper variation, and control-reversal frequency for the baseline, S&C, and the gripper-excluded variant. The outcome is unambiguous and runs against the regularizer's intent: **S&C roughly triples trajectory jerk** (total jerk RMS 1.69 ± 0.09 vs. 0.59 ± 0.07 for the baseline), doubles normalized action variation (0.91 vs. 0.39), increases control reversals by 42% (1054 vs. 741 per episode), and increases gripper variation 2.8 × (182.8 vs. 66.3). Excluding the gripper dimension from the penalty restores gripper variation to the baseline level (70.3 vs. 66.3) while leaving overall jerk essentially unchanged (1.68), so the jerk degradation originates in the continuously penalized dimensions rather than the gripper alone. In short, the smoothness objective made the executed trajectories substantially *less* smooth while also lowering task success—the clearest evidence in this study that the regularizer, as formulated, harms the policy rather than regularizing it usefully.

#### 6.6.1 Compute and latency accounting

LoRA adaptation trains 2.97M of 450M parameters (0.66%). Per-configuration training cost is approximately 4 GPU-hours for 30K steps (0.48 s per update, data-loading bound, identical for baseline and S&C) on the seeded-comparison hardware, and ≈44 GPU-hours for the 100K-step baseline used in the budget sweep. Evaluation costs 2.5–3 GPU-hours per 500-episode cell; peak training memory is 7.3 GB for the baseline and statistically indistinguishable for S&C (the auxiliary head adds < 0.1% parameters). Rollout step rates (Table 7) show the temporal ensemble costs a 2.3–2.4 × slowdown, because it recomputes the policy at every step instead of once per *k* = 8 steps; this is the explicit price of its +17 to +31 pp gains.

### 6.7 Budget sweep (RQ4)

To separate “the regularizer is harmful” from “the regularizer has not converged,” we sweep the training budget with checkpoint-level evaluation on LIBERO-Spatial (seed 42; Figure 6). At 10K steps both configurations are weak (baseline 7.6% vs. S&C 2.0%; Object 9.6 vs. 2.2%). At 30K the gap is maximal (Table 8). Beyond 30K the baseline improves and saturates (external checkpoint evaluations: 14.8% at 20K, 21.6% at 60K, 19.0% at 80K, 21.0% at 100K), whereas S&C stays at or near zero at every measured checkpoint (2.0 → 0.0 → 0.8 → 0.0 → 0.0% through 100K). The deficit therefore *widens and then persists* with budget; it does not close, which rules out insufficient optimization as the sole explanation for the tested configuration range. Figure 7 provides a visual summary of the three-seed pivotal comparison across the four LIBERO suites.

**Figure 6:**
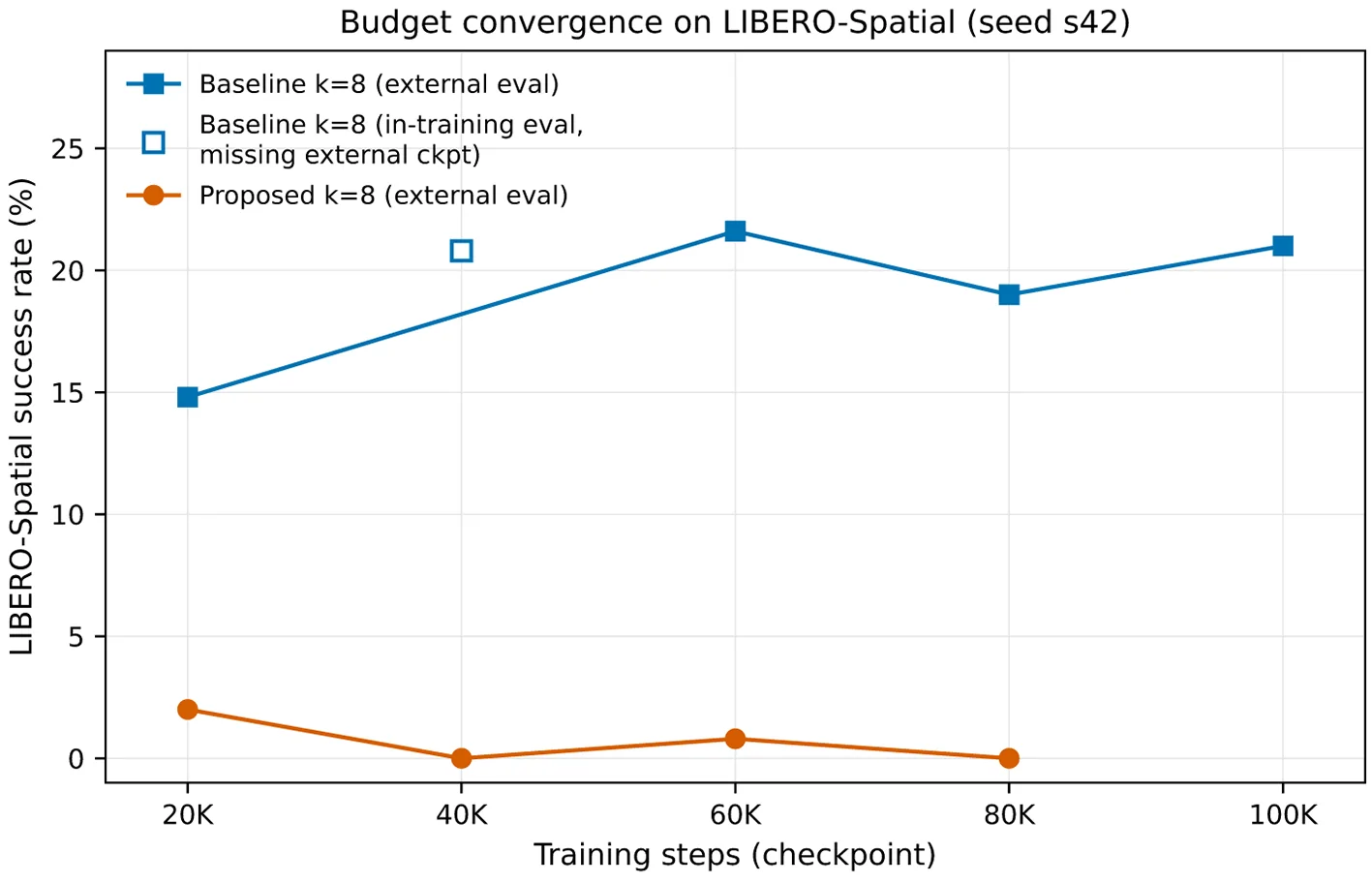
Budget sweep on LIBERO-Spatial (seed 42): suite-level success rate at evaluated checkpoints from 10 to 100K steps. The fixed-chunk baseline improves and saturates; the S&C configuration remains at or near zero at every checkpoint beyond 10K.

**Figure 7:**
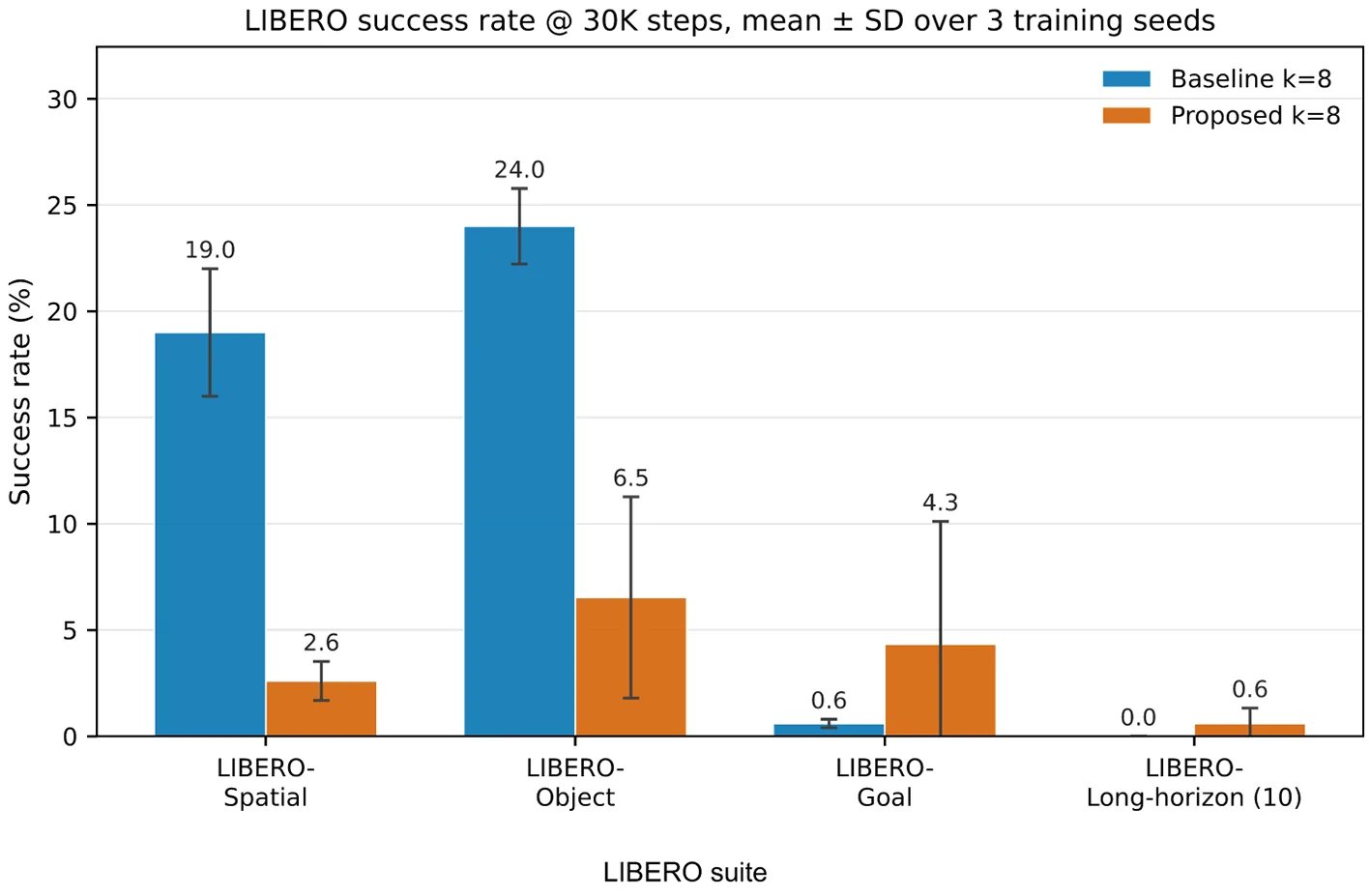
Suite-level success rates (mean ± SD over three training seeds; 500 episodes per cell) for the pivotal comparison. SmolVLA+LoRA+S&C is significantly below the matched *k* = 8 baseline on LIBERO-Spatial and LIBERO-Object (exact sign-flip *p* < 0.005, *r*_rb_ ≤ −0.96); on LIBERO-Goal and LIBERO-10 both configurations are near floor and the difference is not significant.

**Table 8:** Three-seed matrix of the pivotal comparison (500 episodes per cell).

	SmolVLA+LoRA+S&C				**Fixed-chunk** ***k*** = 8			
**Suite**	**42**	**123**	**999**	**Mean** ±**SD**	**42**	**123**	**999**	**Mean** ±**SD**
Spatial	3.6	2.4	1.8	2.6 ± 0.9	19.0	22.0	16.0	19.0 ± 3.0
Object	12.0	3.8	3.8	6.5 ± 4.7	25.4	24.6	22.0	24.0 ± 1.8
Goal	1.0	1.0	11.0	4.3 ± 5.7	0.6	0.4	0.8	0.6 ± 0.2
LIBERO-10	1.4	0.4	0.0	0.6 ± 0.7	0.0	0.0	0.0	0.0 ± 0.0
Macro-4	4.5	1.9	4.2	3.5 ± 1.4	11.3	11.8	9.7	10.9 ± 1.1

Every suite–seed cell is present for both methods, including LIBERO-10 seed 123, which was missing from the earlier submission. mean±SD uses the sample standard deviation over the three seeds, computed per suite and for the macro.

## 7 Discussion

### 7.1 Why the outcome is negative in the tested configuration range

The component ablations localize the failure to the smoothness term: the chunk-aware auxiliary loss alone is approximately neutral, the smoothness loss alone reproduces the collapse, and the primary action-token loss of the full method plateaus roughly 0.28 higher than the baseline's, so the smoothness gradient measurably competes with action prediction rather than acting as a benign regularizer. The executed-action measurements sharpen this conclusion: the regularized policy's trajectories have ≈2.9 × the baseline's jerk and 2.8 × its gripper variation, so the penalty did not even produce smoother outputs—it degraded the very quantity it was meant to improve, most plausibly by biasing the differentiable expected action away from well-calibrated argmax decoding while the policy still executes argmax at runtime. The budget sweep shows that the deficit does not close through 100K steps, which argues against insufficient optimization as the sole explanation within the tested range. The gripper-excluded variant restores gripper variation but not overall jerk, so the failure is not reducible to the binary gripper dimension. These attributions remain ablation- and measurement-based: gradient-level mechanisms and the expectation–argmax mismatch are unmeasured candidate contributors.

### 7.2 What this means for low-cost VLA research

Four takeaways follow. First, training-time regularization claims should be accompanied by matched component ablations *and* by measurements of the regularized quantity itself; here the ablations localized the failure and the jerk measurements reversed the intended interpretation. Second, single-budget studies cannot separate “the regularizer hurts” from “the regularizer has not converged”; the checkpoint-level sweep changed our own earlier interpretation, which had attributed the failure to insufficient optimization. Third, training-time chunk-aware representation learning and inference-time adaptive chunk scheduling must be named and analyzed separately; the retired “adaptive_k8” naming obscured that the runtime chunk length was fixed. Fourth, at small budgets the cheapest strong baseline is inference-side: temporal ensembling delivered the largest gains in this study with zero training, at a quantified 2.3 × inference cost, whereas the training-side regularizer consumed training compute to move every metric in the wrong direction.

### 7.3 Neurorobotics context

The study is fully simulation-based, and its conclusions are restricted to the tested configuration, budget, and benchmark; it does not establish claims about assistive grasping, rehabilitation support, or clinical relevance. For small labs building toward such applications, the practical reading is that, in the tested regime, engineering effort spent on the base imitation-learning pipeline (more demonstrations, suitable fixed chunk sizes) appears better allocated than effort spent on the tested auxiliary losses. Whether the conclusion transfers to real robots, other backbones, or multimodal sensor feedback (Ud Din et al., 2026) is untested.

### 7.4 Qualitative failure illustration

Figure 5 illustrates one failure of the S&C configuration: the policy stalls during approach without completing the grasp, qualitatively consistent with over-regularization of discrete actions. Because no failure taxonomy was annotated across the evaluation episodes, we present this as an illustrative example only and make no claim about the frequency of any failure mode.

### 7.5 Implications for reliability-oriented modeling

The diagnostic study motivates an explicit failure-analysis viewpoint for low-cost VLA post-training: rather than reporting only headline success rates, it decomposes the negative outcome into optimization-budget, classifier-convergence, and over-regularization factors that are individually checkable. Adopting this diagnostic posture is recommended for any action-side regularization claim evaluated under a constrained compute budget.

## 8 Limitations

The study has the following limitations, organized by category.

### 8.1 Compute and experimental design

1. **Compute scale**. The main comparison uses 30,000 steps; the budget sweep extends to 100,000 steps for LIBERO-Spatial (seed 42) only. Extrapolating to the 2,000,000-step budget of the original SmolVLA paper is out of scope.
2. **Training-seed variance**. The pivotal configurations are each trained over three independent seeds and reported as mean ± sample standard deviation (Table 8); the remaining configurations (*k*∈{1, 2, 4}, component ablations, gripper-excluded and ensembling variants) are single-seed (42) and should be read accordingly. Seed-level variance is non-trivial in some cells (e.g., S&C Object spans 3.8%–12.0%), which the three-seed reporting of the pivotal comparison covers but the single-seed rows do not.
3. **Train/validation/test split and cross-validation**. Following the LIBERO benchmark design, the provided demonstration set is used for training and the evaluation episodes serve as a held-out test set, and no separate validation split is used for hyperparameter selection. Two standard procedures were not applied in this submission and remain future work: (a) a three-fold partition into training, validation, and hold-out test sets to control for model-selection bias; and (b) *k*-fold cross-validation to obtain performance-metric distributions for inter-model comparison at larger compute budgets.

### 8.2 Sensing and perception

1. **Sensing-pipeline robustness (brief)**. The experiments use the observation modalities exposed by LIBERO and do not model sensor-calibration drift or multimodal synchronization; real deployments would need automatic recalibration and continuous-time synchronization procedures (Kim and Ha, 2023; Furgale et al., 2013). Because no sensing variation is studied here, we keep this limitation brief and refer to the cited calibration literature for details.

### 8.3 Simulation-to-reality transfer

1. **Simulation-only evaluation**. No real-robot deployment is included. The intrinsic discrepancy between simulated and real-world physics is an open challenge in VLA for robotic manipulation (Ud Din et al., 2026). Two specific gaps are acknowledged: (i) the sim-to-real distribution shift in visual appearance, contact dynamics, and actuator response means that performance measured in LIBERO may not transfer directly to a physical robot; and (ii) mechanistic strategies to minimize this gap—such as domain randomization, system identification, or learned domain adaptation—are not explored in this work. Rather than relying solely on data-generation systems aimed at synthesizing large-scale demonstrations (Shukor et al., 2025), future work should investigate mechanistic approaches to bridging the simulation-to-reality gap.
2. **Inference-time smoothing coverage**. Temporal ensembling of overlapping chunk predictions is measured in this revision (Table 4, Panel C); oracle chunk scheduling, low-pass action filtering, and Kalman smoothing remain unmeasured and are future work.
3. **Limited sensory scope**. The experiments use the observation modalities exposed by LIBERO. The method does not yet integrate tactile sensing, haptic feedback, force feedback, or high-rate proprioception. Future multimodal versions should explicitly estimate inter-sensor temporal offsets or use causal policy-input reconstruction before fusing visual, proprioceptive, tactile, and haptic streams.

## 9 Conclusion

This study tested whether a smoothness regularizer and a chunk-aware auxiliary classifier, added to a SmolVLA+LoRA baseline, improve language-conditioned manipulation at a 30,000-step budget realistic for a small lab. Under a unified 500-episode-per-cell protocol on four LIBERO suites, with both pivotal configurations trained over three seeds, the configuration (SmolVLA+LoRA+S&C) does not improve over the matched fixed-chunk baseline: the macro success rate is 3.5%±1.4 vs. 10.9%±1.1, and the pre-specified exact sign-flip permutation tests localize significant deficits to LIBERO-Spatial and LIBERO-Object. Matched component ablations attribute the deficit to the smoothness loss, the gripper-excluded variant recovers part of it, and a 10–100K checkpoint sweep shows the gap does not close with budget. The evidence supports one bounded conclusion: *for the tested configuration, budgets, and benchmark, the added action-side losses are not competitive with a well-tuned fixed chunk*. It does not support the broader claim that action-side regularization is generally not cost-effective.

The revision contributes the corrected statistical methodology (exact sign-flip permutation tests, recentered bootstrap, Holm-corrected exploratory family, matched-pairs rank-biserial effect sizes, hierarchical seed-level uncertainty), the fully specified and audited pseudo-label construction, matched component ablations, trajectory-smoothness and compute-cost measurements, an in-study temporal-ensembling comparator, and the budget sweep. These materials provide a reproducible basis for follow-up studies of action-side supervision at other budgets and on other backbones.

## Data Availability

Publicly available datasets were analyzed in this study. This data can be found here: the datasets analyzed in this study are publicly available. The LIBERO benchmark and datasets are available from the official LIBERO repository. Additional implementation details and derived experimental metadata are available from the corresponding author upon reasonable request.

## Mathematical definitions of evaluation metrics

For reproducibility, this appendix provides formal definitions of all evaluation metrics.

**Success Rate** **($\hat{p}$)**. For a task *t* evaluated over *n*_*t*_ episodes:

$$\begin{array}{l} {\hat{p}}_{t}=\frac{1}{n_{t}}\overset{n_{t}}{\underset{i=1}{\sum }}⊮\left[s_{t,i}=\text{success}\right], \end{array}$$
(a1)

where *s*_*t, i*_ is the binary outcome of episode *i* for task *t*.

**Macro Success Rate**.

$$\begin{array}{l} {\hat{p}}_{\text{macro}}=\frac{1}{M}\overset{M}{\underset{t=1}{\sum }}{\hat{p}}_{t}. \end{array}$$
(a2)

**Wilson 95% Confidence Interval**.

$$\begin{array}{l} \left[{\hat{p}}_{\text{low}},{\hat{p}}_{\text{high}}\right]=\frac{\hat{p}+\frac{z^{2}}{2n}\pm z\sqrt{\frac{\hat{p}\left(1-\hat{p}\right)}{n}+\frac{z^{2}}{4n^{2}}}}{1+\frac{z^{2}}{n}}, \end{array}$$
(a3)

where *z* = 1.96.

**Exact Sign-Flip Permutation Test (primary)**. For paired differences *d*_*t*_, *t*∈{1, …, 10}, enumerate all sign patterns *s*∈{−1, +1}^10^:

$$\begin{array}{l} p=\frac{1}{2^{10}}\underset{s}{\sum }⊮\left[\left|\text{mean}\left(s⊙d\right)|\geq |\text{mean}\left(d\right)\right|\right]. \end{array}$$
(a4)

**Recentered Bootstrap (null) and Percentile CI**. For the null *p*-value, resample *B* = 10, 000 times from the centered differences $d_{t}-\bar{d}$ and compute $p=\frac{1}{B}\underset{b}{\sum }⊮\left[\left|{\bar{d}}^{*\left(b\right)}|\geq |\bar{d}\right|\right]$. For the confidence interval, resample the uncentered *d*_*t*_ and take the 2.5th/97.5th percentiles of ${\bar{d}}^{*}$.

**Matched-Pairs Rank-Biserial Correlation**. With *W*^+^ and *W*^−^ the sums of positive and negative signed ranks over the non-zero differences,

$$\begin{array}{l} r_{\text{rb}}=\frac{W^{+}-W^{-}}{W^{+}+W^{-}}. \end{array}$$
(a5)

**Wilcoxon Signed-Rank Test (exploratory, Holm-corrected)**.

$$\begin{array}{l} W=\overset{M}{\underset{t=1}{\sum }}\text{sgn}\left(d_{t}\right)\cdot R_{t}, \end{array}$$ (a6)

where *R*_*t*_ is the rank of |*d*_*t*_| among non-zero absolute differences; zero differences are dropped and their count reported. Exact mode is used when no ties/zeros and *n* ≤ 25.

**Friedman Test**.

$$\begin{array}{l} \chi_{F}^{2}=\frac{12M}{K\left(K+1\right)}\left(\overset{K}{\underset{j=1}{\sum }}{\bar{R}}_{j}^{2}-\frac{K{\left(K+1\right)}^{2}}{4}\right). \end{array}$$
(a7)

## References

[B1] Bousmalis K. Trieu G. Zeng A. Hausman K. Ichter B. Sushkov I. . (2024). RoboCat: a self-improving generalist agent for robotic manipulation. Trans. Mach. Learn. Res.

[B2] Brohan A. Brown N. Carbajal J. Chebotar Y. Chen X. Chociej K. . (2023a). “RT-2: vision-language-action models transfer web knowledge to robotic control,” in Proceedings of the 7th conference on robot learning (CoRL) (Atlanta, GA).

[B3] Brohan A. Brown N. Carbajal J. Chebotar Y. Dabis J. Finn C. . (2023b). “RT-1: robotics transformer for real-world control at scale,” in Robotics: science and systems (RSS) (Daegu). doi: 10.15607/RSS.2023.XIX.025

[B4] Chen L. Lu K. Rajeswaran A. Lee K. Finn C. Abbeel P. (2021). “Decision transformer: reinforcement learning via sequence modeling," in *Advances in neural information processing systems (NeurIPS)*.

[B5] Chi C. Feng S. Du Y. Xu Z. Cousineau E. Burchfiel B. . (2023). “Diffusion policy: visuomotor policy learning via action diffusion,” in Proceedings of robotics: science and systems (Daegu). doi: 10.15607/RSS.2023.XIX.026

[B6] Demšar J. (2006). Statistical comparisons of classifiers over multiple data sets. J. Mach. Learn. Res. 7, 1–30.

[B7] Driess D. Xia F. Sajjadi M. S. M. Lynch C. Chowdhery A. Ichter B. . (2023). “PaLM-E: an embodied multimodal language model,” in Proceedings of the 40th international conference on machine learning (ICML) (Honolulu, HI).

[B8] Friedman M. (1937). The use of ranks to avoid the assumption of normality implicit in the analysis of variance. J. Am. Stat. Assoc. 32, 675–701. doi: 10.1080/01621459.1937.10503522

[B9] Furgale P. Rehder J. Siegwart R. (2013). “Unified temporal and spatial calibration for multi-sensor systems,” in IEEE/RSJ international conference on intelligent robots and systems (IROS) (Tokyo: IEEE). doi: 10.1109/IROS.2013.6696514

[B10] Hu E. J. Shen Y. Wallis P. Allen-Zhu Z. Li Y. Wang S. . (2022). “LoRA: low-rank adaptation of large language models,” in International conference on learning representations (ICLR).

[B11] James S. Ma Z. Arrojo D. R. Davison A. J. (2020). RLBench: the robot learning benchmark & learning environment. IEEE Robot. Autom. Lett. 5, 3019–3026. doi: 10.1109/LRA.2020.2974707

[B12] Jang E. Ichter B. Khansari M. Kappler D. Quillen D. Levine S. (2021). “BC-Z: zero-shot task generalization with robotic imitation learning,” in Proceedings of the 5th conference on robot learning (CoRL) (London).

[B13] Jiang Y. Gupta A. Zhang Z. Wang G. Dou Y. Chen Y. . (2023). “VIMA: general robot manipulation with multimodal prompts,” in Proceedings of the 40th international conference on machine learning (ICML) (Honolulu, HI).

[B14] Khazatsky A. Pertsch K. Nair S. Balakrishna A. Dasari S. He P. . (2024). “DROID: a large-scale in-the-wild robot manipulation dataset,” in Robotics: science and systems (RSS) (Delft).

[B15] Kim J.-Y. Ha J.-E. (2023). Automatic extrinsic calibration of a camera and a 2D LiDAR with point-line correspondences. IEEE Access 11, 76904–76912. doi: 10.1109/ACCESS.2023.3298055

[B16] Kim M. J. Pertsch K. Karamcheti S. Xiao T. Balakrishna A. Nair S. . (2024). “OpenVLA: an open-source vision-language-action model,” in Proceedings of the 8th conference on robot learning (CoRL) (Munich).

[B17] Liu B. Zhu F. Zhang C. Song Y. Zhang Z. Jiang X. . (2023). “LIBERO: benchmarking knowledge transfer for lifelong robot learning,” in Advances in neural information processing systems (NeurIPS), datasets and benchmarks track (New Orleans, LA). doi: 10.52202/075280-1939

[B18] Lynch C. Sermanet P. (2021). “Language conditioned imitation learning over unstructured data,” in Proceedings of robotics: science and systems XVII. doi: 10.15607/RSS.2021.XVII.047

[B19] Mandlekar A. Xu D. Wong J. Nasiriany S. Wang C. Kulkarni R. . (2022). “What matters in learning from offline human demonstrations for robot manipulation,” in Proceedings of the 5th conference on Robot learning, Vol. 164 (London: PMLR), 1678–1690.

[B20] Mandlekar A. Zhu Y. Garg A. Booij J. Fei-Fei L. Savarese S. (2018). “ROBOTURK: a crowdsourcing platform for robotic skill learning through imitation,” in Proceedings of the 2nd conference on robot learning (CoRL).

[B21] Nair S. Rajeswaran A. Kumar V. Finn C. Gupta A. (2022). “R3M: a universal visual representation for robot manipulation,” in Proceedings of the 6th conference on robot learning (CoRL) (Auckland).

[B22] Octo Model Team Ghosh, D. Walke H. Pertsch K. Black K. Mees O. . (2024). “Octo: an open-source generalist robot policy,” in Robotics: science and systems (RSS) (Delft).

[B23] Padalkar A. Poole A. Gritsenko A. Singh L. Kalakrishnan M. Dwibedi D. . (2024). “Open X-embodiment: robotic learning datasets and RT-X models,” in IEEE international conference on robotics and automation (ICRA) (Yokohama: IEEE).

[B24] Raspopovic S. (2021). Neurorobotics for neurorehabilitation. Science 373, 634–635. doi: 10.1126/science.abj5259 34353946

[B25] Reed S. Zolna K. Parisotto E. Colmenarejo S. G. Novikov A. Barth-Maron G. . (2022). A generalist agent. Trans. Mach. Learn. Res.

[B26] Shafiullah N. M. M. Cui Z. Altanzaya A. Pinto L. (2022). “Behavior transformers: Cloning *k* modes with one stone,” in Advances in neural information processing systems (NeurIPS) (New Orleans, LA). doi: 10.52202/068431-1668

[B27] Shridhar M. Manuelli L. Fox D. (2021). “CLIPort: what and where pathways for robotic manipulation,” in Proceedings of the 5th conference on robot learning (CoRL) (London).

[B28] Shukor M. Aubakirova D. Capuano F. Kooijmans P. Palma S. Zouitine A. . (2025). SmolVLA: a vision-language-action model for affordable and efficient robotics. arXiv. [preprint]. arXiv:2506.01844. doi: 10.48550/arXiv.2506.01844

[B29] Ud Din M. Akram W. Saad Saoud L. Rosell J. Hussain I. (2026). Multimodal fusion with vision-language-action models for robotic manipulation: a systematic review. Inf. Fusion. 129:104062. doi: 10.1016/j.inffus.2025.104062

[B30] Walke H. Black K. Zhao T. Z. Vuong Q. Zheng C. Hansen-Estruch P. . (2023). “BridgeData V2: a dataset for robot learning at scale,” in Proceedings of the 7th conference on robot learning (CoRL) (Atlanta, GA).

[B31] Wilcoxon F. (1945). Individual comparisons by ranking methods. Biometrics Bull. 1, 80–83. doi: 10.2307/3001968 18903631

[B32] Zeng A. Song S. Lee J. Rodriguez A. Funkhouser T. (2020). “Transporter networks: rearranging the visual world for robotic manipulation,” in Proceedings of the 4th conference on robot learning (CoRL) (Cambridge, MA).

[B33] Zhao T. Z. Kumar V. Levine S. Finn C. (2023). “Learning fine-grained bimanual manipulation with low-cost hardware,” in Robotics: science and systems (RSS) (Daegu). doi: 10.15607/RSS.2023.XIX.016

[B34] Zhu Y. Liu R. Huang S. Weng J. Gong R. Xiang Z. . (2026). A bioinspired multifunctional adhesive-tactile E-Skin enabled for adaptive grasping and slip detection. ACS Appl. Mater. Interfaces. 18, 15635–15646. doi: 10.1021/acsami.6c00064 41785156

